# The Antidepressant Effect of Ketamine Is Dampened by Concomitant Benzodiazepine Medication

**DOI:** 10.3389/fpsyt.2020.00844

**Published:** 2020-08-28

**Authors:** Veronika Andrashko, Tomas Novak, Martin Brunovsky, Monika Klirova, Peter Sos, Jiri Horacek

**Affiliations:** ^1^Clinical Research of Mental Disorders, National Institute of Mental Health, Klecany, Czechia; ^2^Third Faculty of Medicine, Charles University, Prague, Czechia

**Keywords:** ketamine, depression, clinical effect, antidepressant, concomitant medication, benzodiazepines

## Abstract

The rapid antidepressant effect of ketamine has become a breakthrough in the research and treatment of depression. Although predictive and modulating factors of the response to ketamine are broadly studied, little is known about optimal concurrent medication protocols. Concerning gamma-aminobutyric acid neurotransmission being a shared target for both ketamine and benzodiazepines (BZD), we evaluated the influence of BZD on the antidepressant effect of a single ketamine infusion in depressed patients. Data from 47 patients (27 females) with major depression (MADRS ≥ 20, ≥ 1 prior nonresponse to antidepressant treatment in current episode) who participated in two previous studies (EudraCT Number: 2009-010625-39 and 2013-000952-17) entered the analysis. All of the subjects were given an infusion of a subanesthetic dose of racemic ketamine (0.54 mg per kg) as an add-on medication to ongoing antidepressant treatment. Thirteen patients (28%) reached ≥ 50% reduction in MADRS within one week after ketamine administration. Nineteen (40%) patients took concomitant benzodiazepines on a daily basis. The doses of BZDs were significantly higher in nonresponders (p=0.007). ROC analysis distinguished responders from nonresponders by a criterion of >8mg of diazepam equivalent dose (DZ equivalent) with a sensitivity of 80% and a specificity of 85% (p<0.001). RM-ANOVA revealed a different time pattern of response to ketamine between the BZD+ (>8mg of DZ equivalent) and BZD− (≤8mg of DZ equivalent) groups, with a significantly worse outcome in BZD+ on day 3 (p=0.04) and day 7 (p=0.02). The results of the study indicate that concomitant benzodiazepine treatment in higher doses may attenuate ketamine’s antidepressant effect. The pathophysiological, clinical and methodological implications of this finding should be considered in future research and ketamine treatment.

## Introduction

The rapid antidepressant effect of ketamine, first published in 2000 (1) and further abundantly replicated (2), has become a breakthrough in the research and treatment of depression, including novel insight in its mechanisms of antidepressant effect (3, 4). Briefly, the rapid improvement of the depressive mood induced by ketamine may be mediated by the induction of the multiple processes involved in neuroplasticity, with synaptogenesis being a reputable explanation for its antidepressant effect (5–8). These mechanisms are allegedly triggered through ketamine’s antagonism of N-methyl-d-aspartate (NMDA) receptors on gamma-aminobutyric acid (GABA) interneurons and consequent activation of α-amino-3-hydroxy-5-methyl-4-isoxazolepropionic acid (AMPA) receptors (6, 9). Ketamine in subanesthetic doses is considered an auspicious treatment option, particularly in treatment-resistant depression (10).

However, several knowledge gaps are still to be remediated, including the precise methodology of treatment timing, frequency of administration and the effect of concomitant medication (11). In particular, the question of benzodiazepine (BZD) medication in depressed patients receiving ketamine is important for several reasons. Firstly, BZDs represent very commonly prescribed psychotropics (12, 13) and, although not recommended by guidelines (14, 15), are often added to antidepressants in initial depression treatment. In a cohort study, up to 12.5% of depressed patients initiating antidepressant treatment simultaneously started BZD therapy, with 12.3% of them becoming long-term BZD users later on (16). Several other studies have proven that several patients treated for depression fail to withdraw from BZD even after the acute treatment period (17–19), despite the fact that they may contribute to treatment resistance *per se* in these patients (20). Secondly, BZDs act as GABA-A receptor agonists, allosterically increasing the inhibitory tone of GABA-interneurons (21, 22) and may, thereby, interfere with the therapeutic effect of ketamine in light of ketamine’s blockage of NMDA receptors on the identical population of GABAergic interneurons (3, 9).

This interaction was documented in animal experimental studies, contributing to the understanding of both its pharmacodynamics and the behavioral effects of ketamine. Specifically, diazepam selectively blocked ketamine’s influence on the limbic system metabolism (23) and inhibited ketamine-induced hyperlocomotion (24) in rodents. Moreover, the more than five decades of experience in anesthesiology has documented that BZDs constrict ketamine’s psychotomimetic side effects during general anesthesia (25, 26). These clinical experiences together with the opposite effects of ketamine and BZD on GABAergic interneurons suggest that BZD could negatively interfere with ketamine’s antidepressant effect and the treatment outcome. This assumption has been supported by one case report (27) and two case series suggesting the modulatory influence of BZDs on ketamine’s antidepressant effect (28, 29).

In this study, we aimed to assess the influence of BZD on the antidepressant effect of a single ketamine infusion on a large sample of patients with depression, and to elucidate the eventual dose-dependence of this interaction.

## Materials and Methods

### Subjects and Study Design

We included 47 inpatients from two consecutive single-site, randomized, placebo-controlled, double-blind, cross-over trials between 2010 and 2015 and regarding the research question and purpose of this analysis, we utilized the data from the active branch only. The results of the first study have been previously published (30). All of the subjects were 18 to 65 years old inpatients, diagnosed with recurrent or a single episode of Major Depressive Disorder (MDD) according to DSM-IV criteria (31), confirmed also by The Mini-International Neuropsychiatric Interview—M.I.N.I. (32), Czech version 5.0.0. The inclusion criteria were as follows: Montgomery-Åsberg Depression Rating Scale (MADRS) (33) score of 20 and more, at least one prior nonresponse to adequate antidepressant treatment in a current major depressive episode (MDE), and being on a stable dose of antidepressants (monotherapy or combination) for a minimum of four weeks prior to ketamine administration (and 7 days after ketamine infusion). The exclusion criteria were as follows: any suicide risk assessed by clinical examination, current psychiatric comorbidity on Axis I and II (less than 6 months prior to enrolment in the study), serious unstable medical or neurological illness, monoamine oxidase inhibitors and treatment augmentation by lamotrigine, lithium, antipsychotics and a lifetime history of a psychotic disorder or symptoms in first- or second-degree relatives. All of the patients underwent a physical examination, routine blood tests, electrocardiogram, urinalysis, and urine toxicology before inclusion in the study.

The patients taking BZDs before the study were allowed to remain on an unchanged dose during the trial. For emergency purposes (severe anxiety, tension, or agitation), an increased dose of ongoing BZDs or a single dose of BZDs (in BZD nonusers) up to 20 mg of oxazepam per day or its equivalent could be administered. The protocol also had presupposed the administration of parenteral BZDs as emergency medication in the event of extreme anxiety or agitation as an adverse reaction during the infusion of ketamine.

Patients using BZDs regularly (regardless of dose) during the study period (7 days before the infusion and for 7 days after ketamine infusion) were defined as BZD users and patients without regular anxiolytic medication or receiving BZDs not more than twice per week, were defined as BZD nonusers. For the purpose of the study, the dosage of BZDs was adjusted to estimated diazepam equivalent (DZ equivalent) as a reference (e.g. 10 mg DZ equivalent = 20 mg of oxazepam or 0.75 mg of alprazolam/clonazepam) (34).

All of the patients provided an informed written consent. Both source studies were registered in the European Clinical Trials Database (EudraCT Number: 2009-010625-39 and 2013-000952-17), approved by the Ethical committee of Prague Psychiatric Centre/National Institute of Mental Health, Czech Republic and performed in accordance with the ethical standards from the Declaration of Helsinki 1975, revised Hong Kong 1989.

### Treatment and Assessment

All of the patients received an intravenous infusion of racemic ketamine hydrochloride (Calypsol, Gedeon Richter Plc., Czech Republic), delivered *via* an infusion pump (ID 20/50, Polymed medical CZ Ltd) at fixed time from 8 a.m. to 8:30 a.m. Ketamine was administered as a loading dose of 0.27 mg/kg for the first 10 min, followed by a maintenance infusion of 0.27 mg/kg within 20 min. This dosing schedule was calculated with respect to the pharmacokinetics of ketamine (35, 36) in order to produce stable ketamine blood levels and the total dose applied was the same as in the majority of antidepressant trials of intravenous ketamine (2).

The severity of depressive symptoms was assessed by the MADRS at the baseline (day before the infusion), and 24 h, 72 h, and 7 days after the infusion. Subjective scales (Beck Depression Inventory (BDI) and Beck Anxiety Inventory (BAI) were administered at the same time points. Response to treatment was defined as a MADRS reduction of at least 50% from the baseline at any visit during the 1-week follow-up period.

Blood sampling was performed in order to evaluate serum levels of ketamine and its first metabolite norketamine (2 ml of venous blood 5 min before the infusion, 10 and 30 min after the beginning of the infusion). For the assessment of ketamine and norketamine serum levels, we used gas chromatography–mass spectrometry (GS-MS), developed and validated according to international standards (37). The analytical standards of norketamine, ketamine and deuterated ketamine (ketamine-D4) supplied as hydrochlorides from Cerilliant, USA were used. For quantitation, the internal standard method was applied using ketamine-D4. Isolation of analytes from blood serum samples was performed using SPECDAU disks and the analyses were performed with acetyl derivatives using a HP 6890–5973 instrument (Agilent, Germany) operating in THE electron impact single ion monitoring (SIM) mode. The lower limit of quantification (LLOQ) for ketamine was 50 ng/ml and for norketamine 8 ng/ml. The limit of detection (LOD) for ketamine was 20 ng/ml and for norketamine 1 ng/ml (37).

### Statistical Methods

Demographic, clinical, and treatment characteristics including ketamine and norketamine serum levels, fluoxetine dose equivalent of ongoing antidepressants (38), DZ equivalent (34), and proportion of BZD users (using BZDs on a daily basis regardless of dose), between responders (≥50% MADRS score reduction from the baseline at any visit during the one-week follow-up period) and nonresponders were compared using the unpaired t test, Mann-Whitney U test and Fisher’s exact test as appropriate. As the BZD doses were allowed to be adjusted during the study, DZ equivalent was calculated as the mean daily dose from one day before ketamine administration to the end of the follow-up (day −1 to day 7). In a second step, to address the possibility of different treatment outcomes between zero-to-low-dose and high-dose BZD users (rather than between clear-cut defined BZD users and nonusers), we applied a receiver operating characteristic (ROC) curve to determine the cut-off BZD daily dose with the highest combined sensitivity and specificity for distinguishing responders and nonresponders. BZD+ (BZD high-dose users) and BZD– (BZD zero-to-low-dose users) were compared across demographic and clinical parameters using the unpaired t test, Mann-Whitney U test and Fisher’s exact test and then entered into a logistic regression model as a categorical variable to predict the treatment outcome (response), adjusted for baseline depression and anxiety severity (MADRS, BAI) as possible confounders. Repeated measures analysis of variance (RM-ANOVA) with BZD+ and BZD− as a group variable and MADRS scores at four time points (baseline to day 7) as a within subject repeated variable with the degree of freedom corrected for a lack of sphericity, was performed to find a different pattern of response to ketamine within the study period, if present. In the event of a significant group x time interaction, Bonferroni *post hoc* tests was used. All of the tests were two-sided and an exact significance level of 0.05 was adopted. The analyses were performed using Statistica, version 12 [StatSoft, Inc. (2013)] and MedCalc, version 15.11.4 (MedCalc Software, Ostend, Belgium).

## Results

The data obtained from 47 patients (27 (57%) females) aged between 31 and 52 years (mean 43.0 ± 12.3) were entered into the analyses. The mean baseline depression severity measured by MADRS was 24.0 ± 6.3. The mean duration of depression diagnosis was 10.0 ± 10.1 years, whereas the mean duration of current depression episode was 19.3 ± 26.7 weeks. Thirteen patients (28%) were on AD monotherapy and 34 (72%) had a combination of two or more antidepressants. Sertraline and escitalopram (together 36%), venlafaxine (28%), and mirtazapine (17%) were the most frequently used antidepressants in the study patients. The mean AD daily dosage was 54.1 ± 24.5 mg of fluoxetine equivalent (38). Thirteen patients (28%) responded to a single dose of ketamine (≥ 50% MADRS reduction anytime within one week). The responders had significantly lower baseline anxiety (p=0.04) and depression severity levels (p=0.052), otherwise both groups were comparable (**Table 1**).

**Table 1:** Baseline characteristics of responders vs. nonresponders.

Baseline parameters	Responders (n=13)	Nonresponders (n=34)	p value
Age	41.6 ± 13.1	43.5 ± 12.2	0.64^1^
F/M	6/7	21/13	0.51^2^
AD mono/comb	5/8	8/26	0.46^2^
MDD duration (years)	5.9 ± 5.9	11.6 ± 10.9	0.10^3^
Current episode duration (weeks)	10.8 ± 9.4	22.6 ± 30.4	0.13^3^
No hospitalization	2.4 ± 2	2.7 ± 2.4	0.70^3^
Baseline BDI	14.8 ± 5.4	18.9 ± 6.9	0.06^1^
Baseline BAI	14.9 ± 9.1	22.2 ± 11.3	0.04^1^
Baseline MADRS	21.1 ± 6.5	25.0 ± 5.9	0.052^1^
First/recurrent episode	5/8	6/28	0.25^2^
FLX equi	46.9 ± 16.8	56.9 ± 26.5	0.21^1^
DZ equi	7.7 ± 4.5mg	32.1 ± 24.9mg	0.007^3^
Ketamine plasma level at 10 min (ng/ml)	226 ±103	243 ± 114	0.7^1^
Ketamine plasma level at 30 min (ng/ml)	238 ± 70	243 ± 85	0.8^1^
Norketamine plasma level at 10 min (ng/ml)	9 ± 4	15 ± 12	0.1^1^
Norketamine plasma level at 30 min (ng/ml)	62 ± 26	71 ± 26	0.3^1^

FLX equi, fluoxetine dosage equivalent for AD (38); DZ equi, diazepam dosage equivalent for BZD (34); F/M, female/male; AD, antidepressant; MDD, major depressive disorder. ^1^Unpaired t-test, ^2^Fisher’s exact test, and ^3^Mann-Whitney U test were used to compare the groups.

Twenty-one patients have used at least one dose of BZDs during the study period. Two of them required just a single BZD dose due to worsening anxiety (one patient 24 h before and second one day after ketamine administration). Nineteen patients (40% of sample) were taking concomitant BZDs on daily basis upon admission to the study (BZD users) with doses varying from 3.8 to 94.0 mg of DZ equivalent per day. Thirteen (68%) patients were taking clonazepam, five (26%) took alprazolam and one patient had a combination of clonazepam and midazolam. While concomitant BZD medication (of any dose) was equally distributed in the responders (38%) and nonresponders (42%), both groups markedly differed in the BZD doses with significantly higher doses in nonresponders (7.7 ± 4.5 vs. 32.1 ± 24.9 mg of DZ equivalent; p=0.007, **Table 1**).

**Figure 1:**
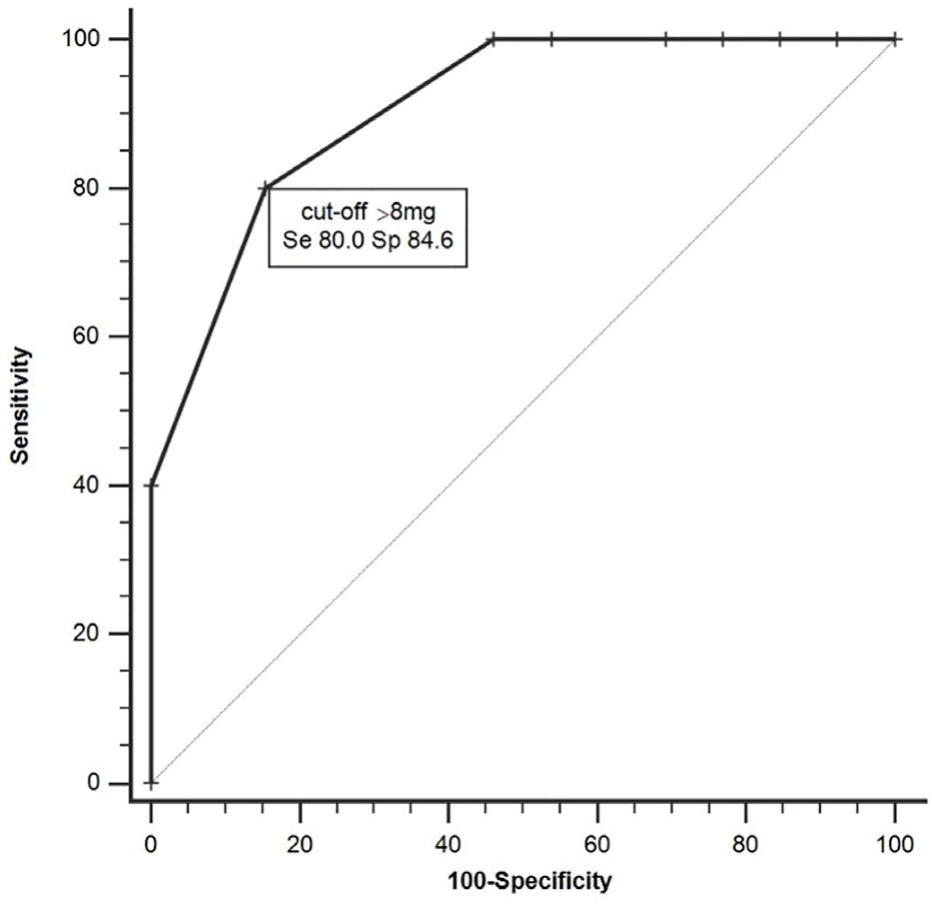
Receiver operating characteristic (ROC) analysis distinguishing responders and nonresponders (AUC=0.91, 95%CI 0.68–0.99, p < 0.001).

**Figure 2:**
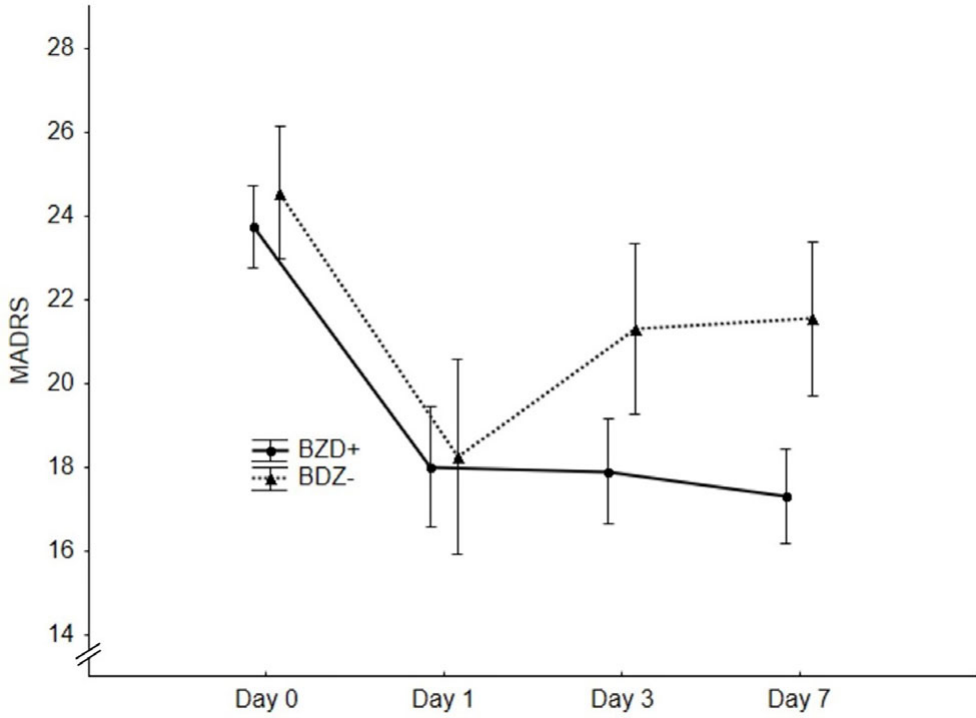
Significantly better outcome in the benzodiazepines (BZD−) group on day 3 (p = 0.04) and day 7 (p = 0.02) revealed by repeated measures analysis of variance (RM-ANOVA).

The ROC analysis distinguished nonresponders from responders by a criterion of >8mg of DZ equivalent with a sensitivity of 80% and a specificity of 84.6% (AUC=0.91, 95%CI 0.68-0.99, p=0.001, **Figure 1**). After dividing the patients into high-dose BZDs users (BZD+, >8mg of DZ equivalent, N=13) and none-to-low-dose BZDs users (BZD-, 0–8mg of DZ equivalent, i.e., up to 0.5mg of clonazepam or alprazolam; N=34), only one patient from the BZD+ group belonged to the responders, while the other responders were from the BZD- group (response rate 8% in BZD+ vs. 35% in BZD-; Fisher’s exact test, p=0.07). Additional logistic regression with a response as a dependent variable adjusted for baseline depression (MADRS score) and anxiety (BAI score) severity as possible confounding factors indicated a higher chance of being a nonresponder in patients on high-dose BZDs after ketamine infusion (OR=9.7, 95%CI 1.2–69.7, p=0.03). Moreover, when MADRS scores between the BZD+ and BZD- group were compared from the baseline to day 7, a different time pattern of response to ketamine was revealed (RM-ANOVA; group x time interaction: F(3,135)=2.98, p=0.03), with a comparable decrease of depressive symptoms 24 h after infusion (Bonferroni *post hoc* test, p=0.53), but a significantly worse outcome in BZD+ on day 3 (p=0.04, Hedges´ g= 0.67) and on day 7 (p=0.02, g= 0.78) (**Figure 2**). Otherwise, the BZD+ and BZD- groups did not differ either in demographic or clinical variables, or in ketamine and its metabolite norketamine plasma levels (**Table 2**).

**Table 2:** Characteristics of high-dose benzodiazepines (BZD) users (BZD+; >8mg of DE) and none-to-low-dose BZD users (BZD-; 0–8mg of DE) during the follow-up period.

Parameters	BZD +(n=13)	BZD -(n=34)	p value
Responders/Nonresponders	1/12	12/22	0.07^1^
Age	41.3 ± 14.2	43.6 ± 11.7	0.57^2^
MDD duration (years)	11.7 ± 14.2	9.4 ± 8.1	0.48^3^
Current episode duration (weeks)	24.3 ± 41.0	17.4 ± 19.3	0.43^3^
Baseline BDI	17.1 ± 7.6	18.1 ± 6.5	0.65^2^
Baseline BAI	21.7 ± 14.2	19.6 ± 9.9	0.56^2^
Baseline MADRS	24.5 ± 8.2	23.7 ± 5.4	0.70^2^
MADRS change baseline vs. day 1	0.2 ± 0.3	0.3 ± 0.3	0.52^2^
MADRS change baseline vs. day 3	0.04 ± 0.43	0.26 ± 0.28	0.04^2^
MADRS change baseline vs. day 7	0.03 ± 0.47	0.30 ± 0.27	0.02^2^
Ketamine plasma level at 10 min (ng/ml)	276 ±148	226 ± 94	0.22^2^
Ketamine plasma level at 30 min (ng/ml)	262 ± 87	235 ± 79	0.36^2^
Norketamine plasma level at 10 min (ng/ml)	18 ±13	12 ± 10	0.1^2^
Norketamine plasma level at 30 min (ng/ml)	80 ± 39	65 ± 28	0.2^2^

^1^Fisher’s exact test, ^2^unpaired t-test, and ^3^Mann-Whitney U test were used to compare the groups.

## Discussion

The main finding of this study is that BZDs attenuate ketamine’s antidepressant effect in patients with MDD. This effect occurred in patients taking higher BZD doses and remained present even when adjusted to baseline anxiety and depression scores and was also not affected by plasma levels of ketamine and its first metabolite norketamine measured during or after the infusion.

Our findings are congruent with two previous case-series reports [pointing lower daily dose of BZDs in ketamine responders among 10 depressed patients (28) or accelerated loss of therapeutic effect in 4 out of 13 subjects (29)] and a case report of a patient with attenuated response to ketamine with concurrent lorazepam (27)] and have pathophysiological and substantial clinical and methodological implications. Firstly, we speculated that BZDs increase the inhibition of pyramidal neurons and, thereby, counteract the inhibition of GABA-interneurons mediated by ketamine-induced antagonism of extrasynaptic NMDA receptors, located foremost at these interneurons (3). This interpretation is in line with the fact that BZDs effectively inhibit the psychotomimetic effect of ketamine during or after general anesthesia (25, 26), especially considering the correlation between the intensity of psychotomimetic or dissociative symptoms during ketamine infusion and its antidepressant effect, documented in our previous study (30), and replicated for an independent sample (39). Interestingly, our results show that BZDs only interfered with a delayed response to ketamine, observed on day 3 and day 7 after the administration, but not in the first 24 h. This fact suggests that BZD-mediated inhibition of ketamine’s antidepressant effect may be related to attenuation of neuroplastic processes, emerging subsequently after the acute effect and after ketamine and its active metabolites are eliminated from the blood. Preclinical data suggested that a ketamine-induced elevation of synaptic proteins (such as synapsin I, PSD95 and GluR1) could be initially observed 2 h after the application and remained increased up to 7 days (8). This observation corresponds with the duration of ketamine’s antidepressant effect in humans (2). Together, a subanesthetic dose of ketamine acutely induces dissociative/psychotomimetic effects by blocking NMDA receptors, followed by an extracellular release of glutamate. However, glutamate then preferentially activates glutamatergic AMPA receptors, inducing their phosphorylation (40, 41) and subsequently triggering activation of protein kinases such as mTOR (mammalian target of rapamycin), promoting an expression of neurotrophic proteins that regulate synaptic growth and remodellation (39, 42). Our data suggest that BZDs may interfere with these downstream signaling steps and, therefore, relate to a sustained antidepressant effect. This assumption should be elucidated by future research addressing the temporal dynamics of BDNF (brain-derived neurotrophic factor) and other neurotrophins in patients with concomitant BZDs.

The fact that BZDs did not influence ketamine’s antidepressant effect in the first 24 h (similar improvement in both groups) may be interpreted by possible initial symptom relief due to a robust acute psychological effect (not related to the antidepressant effect *per se*) linked to psychotropic effect of ketamine infusion. This psychotropic effect of ketamine limits effective study blinding (43, 44), especially when an inactive comparator is used.

Clinically, our results raise discussion on the negative therapeutic impact of concomitant BZD medication in depressed patients, whereas concurrent medication is indeed a modifiable factor, unlike other (e.g. demographic or pharmacokinetic) characteristics in subjects receiving ketamine. Anxious depression has been shown to be associated with a poorer response to antidepressant treatment *per se* (45). Moreover, BZDs have been identified as a possible causative factor for treatment resistance in depressed patients (20). With ketamine being a novel option in treatment-resistant depression, it is likely that a substantial number of patients could be on BZDs at the time of infusion. This should be taken into account especially since the U.S. Food and Drug Administration has recently approved intranasal esketamine as an additional treatment for resistant depression, where the influence of BZD’s may be similar, albeit to our knowledge there is yet no published data examining this consideration.

In addition, ketamine’s effect may also be influenced by antiglutamatergic anticonvulsive drugs used in bipolar depression or as adjunctive therapy in resistant unipolar depression (46). It has been documented that pre-treatment with antiglutamatergic anticonvulsant lamotrigine attenuates ketamine’s acute neuropsychiatric effects (47, 48) as well as its neuroimaging correlates in pharmacological fMRI studies (48, 49). Nevertheless, the interaction between antiglutamatergic anticonvulsants and the antidepressant effect of ketamine was not yet systematically evaluated in MDD patients, apart from an anecdotal report where no difference was found in glutamatergic medication between ketamine responders and nonresponders among 10 patients (28).

Our results should be interpreted in the context of several limitations. First, we utilized retrospective analysis of previously existing data, thus were disabled in designing and controlling the preceding experiment. Second, the study was performed on a relatively small sample size. Third, due to retrospective character of the study, blood levels of BZDs were not available for the analysis and thus we were not able to objectively assess their dose-dependent impact taking into account their pharmacokinetics. Last, the responders and nonresponders in our study differed in baseline anxiety scores (BAI, **Table 1**) with the difference in baseline depression being close to significant. Despite the elimination of these confounding factors by performing additional logistic regression, the small sample size and *post hoc* design may enervate the magnitude of our consideration about BZDs being a solitary determinant. The severity of symptomatology remains relevant especially in the scope of recent studies focusing on the differences in cortical excitation and inhibition in regard to glutamatergic (50) and GABAergic (51, 52) neurotransmission, showing that higher severity and/or resistance of depressive symptomatology is linked to impaired GABAergic functions, especially regarding GABA neurotransmission as a junction target for ketamine and benzodiazepines.

Our findings evoke a substantial methodological implication for future studies with ketamine, suggesting that: a) the results should be replicated together with blood levels of BZDs and b) concomitant medication should be controlled and comprised as an independent factor in effectiveness studies and meta-analyses. Regarding the kinetics of different BZDs, further studies should also address the time period for withdrawal before ketamine infusion.

To conclude, our study indicates that concomitant BZD treatment attenuates ketamine’s antidepressant effect. This fact should be considered in the clinical management of ketamine treatment and future research design.

## Data Availability Statement

The datasets generated for this study are available on request to the corresponding author.

## Ethics Statement

The studies involving human participants were reviewed and approved by Ethical committee of Prague Psychiatric Centre/National Institute of Mental Health, Czech Republic. The patients/participants provided their written informed consent to participate in this study.

## Author Contributions

PS designed and directed the studies and performed the clinical experiments together with MK. JH encouraged VA to investigate the topic. VA wrote the manuscript with input from TN, who performed the statistical calculations. TN, JH, MB, and VA contributed to the interpretation of the results. JH supervised the project and the process of manuscript preparation.

## Funding

This work was supported by: Czech Health Research Council grant number NV18-04-00260, ERDF/ESF project PharmaBrain, Reg. No. CZ.02.1.01/0.0/0.0/16_025/0007444, by grant number LO1611 from the Ministry of Education, Youth and Sports of the Czech Republic under the NPU I program and by MH CZ - DRO (“National Institute of Mental Helth – NIMH, IN: 00023752).

## Conflict of Interest

TN reports that he has received honoraria from KRKA ČR s.r.o. in the past three years.

The remaining authors declare that the research was conducted in the absence of any commercial or financial relationships that could be construed as a potential conflict of interest.
